# A cross-sectional survey exploring the knowledge, experiences and attitudes of Australian pharmacists toward medicinal cannabis

**DOI:** 10.1007/s11096-022-01519-z

**Published:** 2022-11-29

**Authors:** Zeeta Bawa, Bandana Saini, Danielle McCartney, Miguel Bedoya-Pérez, Andrew J. McLachlan, Iain S. McGregor

**Affiliations:** 1 Lambert Initiative for Cannabinoid Therapeutics, The University of Sydney, Sydney, NSW Australia; 2 Brain and Mind Centre, The University of Sydney, Sydney, NSW Australia; 3 Faculty of Science, School of Psychology, The University of Sydney, Sydney, NSW Australia; 4 Sydney Pharmacy School, The University of Sydney, Sydney, NSW Australia; 5 Woolcock Institute of Medical Research, Sydney, NSW Australia

**Keywords:** Cannabidiol (CBD), Cannabinoid, Cannabis, Community pharmacy, Medicinal cannabis, Pharmacists

## Abstract

**Background:**

Australian pharmacists currently dispense a wide range of prescription-only cannabis-based medicines. Recent regulatory changes will expand the role of pharmacists, allowing certain low-dose cannabidiol products to be supplied without a prescription in pharmacies. This harmonises Australia with many other countries where cannabidiol products are readily available to consumers.

**Aim:**

To examine Australian pharmacists’ experience, knowledge and attitudes towards medicinal cannabis and their preparedness to supply over-the-counter low-dose cannabidiol products.

**Method:**

We conducted a cross-sectional study using a 51-item on-line questionnaire that was informed by previous surveys of health professionals and assessed for face validity. Australian pharmacists were recruited to complete the survey between May and December 2021, primarily through professional pharmacy organisations. Pharmacists were included in the final dataset if they completed the demographic characteristics section and at least one additional section of the questionnaire. Data were analysed using descriptive and relational statistical tests.

**Results:**

There were 272 attempts to complete this survey and 217 responses included in the final dataset. Over half of the respondents (60.0%, 130/217) had dispensed at least one medicinal cannabis prescription during their career and 58.5% (127/217) had received at least one medicinal cannabis enquiry in the last fortnight. Only around half (53.9%, 117/217) felt comfortable supplying medicinal cannabis products and fewer (39.3%, 79/201) were confident discussing cannabis-related enquiries. More than half of the respondents (58.7%, 118/201) supported the provision of low-dose cannabidiol products through pharmacies. Two-thirds (67.8%, 80/118) of respondents achieved relatively low scores (< 60%) in the knowledge component of the survey. Most respondents (94.2%, 178/189) endorsed a need for further training in this area.

**Conclusion:**

Australian pharmacists tended to support medicinal cannabis availability and improved access to low-dose cannabidiol products via pharmacies. However, results highlight a need for improved training and education of pharmacists around cannabis-based medicines.

**Supplementary Information:**

The online version contains supplementary material available at 10.1007/s11096-022-01519-z.

## Impact statements

- The legalisation of cannabis-based medicines has created an emerging area of pharmacy practice.
- There is a need to provide pharmacists with further training and education around cannabis-based medicines and over-the-counter cannabidiol products.
- Efforts to increase pharmacists’ knowledge, confidence and competence in this area have the potential to enhance health outcomes for patients using these products.

## Introduction

The term ‘medicinal cannabis’ (MC) is often used to describe cannabis or cannabis-containing products that are used therapeutically to achieve a curative or remedial effect [1]. Cannabidiol (CBD) and delta-9-tetrahydrocannabinol (THC) are the two most-studied cannabis constituents [2]. Scientifically, they are termed “cannabinoids”, reflecting their distinctive chemotype and their interactions with the human endogenous cannabinoid system [3, 4]. THC is renowned for its intoxicating and euphorigenic effects but has also demonstrated efficacy in treating conditions such as chronic pain, chemotherapy-induced nausea and vomiting, and spasticity in multiple sclerosis [2, 5–10]. CBD is a non-intoxicating cannabinoid with a well-established safety profile at therapeutic doses [11–15]. Clinical trials have demonstrated efficacy of CBD in treating epilepsy, anxiety, and psychosis [16–18].

Access to MC products by Australian patients was legalised in November 2016 via specialised schemes that are overseen by the Australian medicines regulator (the Therapeutic Goods Administration, TGA) [19, 20]. Clinicians must obtain TGA approval to prescribe a specific MC product for an individual patient (Special Access Scheme) or patients with a specific condition (Authorised Prescriber Scheme). The resulting prescription is subsequently dispensed through a pharmacy [21–23]. Over the past five years, there has been a rapid rise in demand for MC products with more than 285,000 approvals issued as of September 2022 [24]. Nonetheless, Australian consumers have voiced concerns that access pathways are difficult to navigate and that available products are too expensive [19, 25].

To further improve access, the TGA announced in December 2020, that low-dose, orally administered CBD products (≤ 150 mg daily dose, containing < 1% THC) would be down-scheduled to become available without a prescription as *Pharmacist Only Medicines* [26]. This would enable pharmacists to supply these products to consumers over-the-counter for the short-term management of low-risk indications [27]. This development aligns Australia with North America and Europe, where CBD products are readily accessible to consumers without a doctor’s prescription [19]. At the time of writing, *Pharmacist Only* CBD products are not yet sold in Australia; no manufacturer has registered a relevant product with the TGA. Registration is a lengthy process that involves presenting the TGA with high-quality product efficacy and safety data from clinical trials. Once *Pharmacist Only* CBD products do become registered, Australian pharmacists will likely be met with significant demand for these products [28] as has been the case internationally [29, 30].

The evolving regulatory landscape of MC access requires pharmacy professionals to have specific expertise around cannabis-based medicines. However, given the complicated MC access pathways and the lag in the registration of *Pharmacist Only* CBD products, it is likely that pharmacy professionals have some uncertainty around low-dose CBD product supply. Indeed, a common theme emerging from preceding surveys of pharmacy practitioners globally is an overall lack of knowledge and confidence in providing non-prescription CBD products [31–33]. An exploratory study of pharmacists’ perceptions and experiences around MC found pharmacists had a considerable lack of comfort and preparedness in counselling patients around MC therapy [34]. In Australia, a 2016 semi-structured interview of pharmacists around the role of MC in clinical therapy uncovered a widespread lack of understanding of this drug class [1]. In the six years of legal MC since, there have been no further studies exploring the attitudes, beliefs and knowledge of Australian pharmacists around MC products.

### Aim

The present study investigated the preparedness of the pharmacy profession to supply low-dose *Pharmacist Only* CBD products and more generally, the knowledge, experience, attitudes, and education needs of Australian pharmacists concerning cannabis-based medicines.

### Ethics approval

Approval was granted by the University of Sydney Human Research Ethics Committee on 16 April 2021 (Ref: 2021/149).

## Method

### Participant eligibility and recruitment

An on-line cross-sectional survey was conducted between May and December 2021. Participants met the inclusion criteria if they were registered pharmacists working in an Australian community pharmacy. Australian pharmacy organisations (The Pharmacy Guild of Australia and the Pharmaceutical Society of Australia) assisted with participant recruitment by promoting the survey through their professional education events, social media channels and private mailing groups. This study aimed to recruit 250–300 pharmacists, with this number determined both by sample sizes of our teams’ earlier surveys of health professionals around MC and resourcing constraints [35–37]. As an incentive to complete the survey, pharmacists were given the chance to enter a draw to win one of three Apple watches after survey completion.

### Survey design

The design of the questionnaire (see Online Resource 1 for a full copy) was informed by previous surveys of health professionals around cannabis-based medicines [1, 32, 33, 35, 36]. The questionnaire also uniquely queried Australian pharmacists’ perspectives and knowledge of *Pharmacist Only* CBD products, given the recent legislative changes affecting these products. It contained five sections and a total of 51 items (Table 1) that took ~ 15-minutes to complete. The questionnaire was administered using a secure, web-based platform (REDCap^®^ 12.0.7, 2022, Vanderbilt University). Pharmacists were required to review the Participant Information Statement and complete an online checkbox to confirm informed consent before commencing the questionnaire. An adaptive algorithm and assortment of query formats were used to reduce respondent fatigue and maximise completion rates, including multiple choice, yes-no-unsure, true-false-unsure and 5-Point Likert Scale (e.g., Strongly Agree, Agree, Neutral, Disagree or Strongly Disagree) formats. An option for open-ended comments was also available at the end of the questionnaire. The survey was specifically developed for this study. The survey underwent three rounds of iterative review by pharmacists and academic researchers to achieve face validity.

**Table 1:** Survey design

Section	Query type	Number and format of items
Section 1	Demographic information	Multiple choice (10 items)
Section 2	Experience with supply of medicinal cannabis products	Multiple choice & yes-no-unsure (11 items)
Section 3	Knowledge around medicinal cannabis products	True-false-unsure (13 items)
Section 4	Perspectices on the supply of medicinal cannabis products	5-Point likert scale & multiple choice (11 items)
Section 5	Professional education needs	5-Point likert scale; multiple choice; yes-no-unsure & open text (6 items)

### Data management and analysis

Data were screened for discrepancies such as non-completions and cleaned data were analysed. Participants were included in the final dataset if they completed the first (i.e., Demographic Characteristics Information) section and at least one additional section of the survey. Results were summarised using descriptive statistics (e.g., proportions, medians, ranges), relational analyses (frequency and percentage of valid responses, IBM SPSS Statistics V.24.0 (IBM, U.S.)), correspondence analyses (R V4.1.2 (R Core Team, 2022 [38], function *CA* from the package ‘FactoMineR’ [39]) and Asymptotic Linear-by-Linear Association tests (function *lbl_test* from the package ‘coin’ [40]). Graphs were created using GraphPad Prism V.9.3.1 (350) for Mac (GraphPad Software, La Jolla, California, USA). Open-text comments were grouped by common themes (e.g., perceived benefits and challenges).

Responses to the 5-point Likert scale questions were collapsed into three categories: Agree, Neutral and Disagree. A composite score for knowledge was generated by summing the number of correct responses to the 13 knowledge-based questions. Scores ≥ 60% (≥ eight from a total of 13 questions) were regarded as ‘satisfactory’ and scores < 60% (< eight from a total of 13 questions) were regarded as ‘low.’ While there is an inherent arbitrariness to setting scores [41], knowledge scores ≥ 60% were considered acceptable given that pharmacists were yet to manage any over-the-counter CBD products. A higher level of knowledge would be preferable once these products become registered and widely available.

A series of Asymptotic Linear-by-Linear Association tests were performed to determine independence between ordinal response variables (comfort with supply, confidence, need for training and how supportive pharmacists were of low-dose CBD) and predictive variables (age, gender and experience). Due to multiple tests performed per ordinal response variable, Bonferroni correction was used to adjust α (i.e., 0.05/3 = 0.017). Correspondence analyses were then used to visually represent the significant relationships between variables to determine the strength of the associations.

## Results

A total of 272 individuals initiated the survey, 55 were considered ‘non-completers’ and 217 responses from pharmacists were included in the final dataset. The number of respondents who completed each section was as follows: Experience (n = 217, 100%), Attitudes (n = 201, 92.6%), Professional Educational Needs (n = 189, 87.1%) and Knowledge (n = 118, 54.3%). A technical issue resulted in some respondents (33.4%, 91/272) being unable to complete the Knowledge section of the survey.

### Demographic characteristics

Respondents demographics are summarised in Table 2. Many respondents identified as female (67.7%, 147/217). More than half (52.1%, 113/217) of the participating pharmacists worked in pharmacies within major suburban cities. Half of the respondents (51.2%, 111/217) were located in the most populous Australian state of New South Wales (NSW). The most common age range was 25–34 years (29.5%, 64/217).

**Table 2:** Demographic characteristics of participating pharmacists, n = 217

Characteristics	n = 217	%
Gender identity		
Male	63	29.0
Female	147	67.7
Not provided	7	3.2
Age (years)		
18–24	18	8.3
25–34	64	29.5
35–44	59	27.2
45–54	40	18.4
55–64	28	12.9
65 +	8	3.7
State		
New South Wales	111	51.2
Queensland	40	18.4
Victoria	28	12.9
Western Australia	15	6.9
South Australia	12	5.5
Australian Capital Territory	8	3.7
Tasmania	3	1.4
Northern Territory	0	0
Years of experience		
Less than 1 year	16	7.4
1–5 years	45	20.7
6–10 years	35	16.1
11–15 years	31	14.3
16–19 years	20	9.2
20 or more years	70	32.3
Pharmacy location		
Major metropolitan city centre	39	18.0
Major suburban city centre	113	52.1
Regional town	44	20.3
Rural or remote town	17	7.8
Other	4	1.8

### Pharmacists’ experiences with the supply of medicinal cannabis products

Over half of the respondents (60.0%, 130/217) had dispensed at least one MC prescription during their careers. During the last two months, 39.2% (85/217) had dispensed at least one MC prescription and of these, many (77.6%, 66/85) had dispensed between one and nine MC prescriptions. A small proportion of pharmacists (4.6%, 10/217) had dispensed ≥ 20 MC prescriptions in the last two months. Over half of the respondents (56.7%, 123/217) felt that MC enquiries had risen over the past three months and had received at least one MC enquiry in the past two weeks (58.5%, 127/217).

Just over half of the respondents (53.9%, 117/217), regardless of whether they had supplied MC, felt ‘comfortable supplying MC products and wished to do so in the future.’ The remainder (46.1%, 100/217) felt: ‘neither comfortable nor uncomfortable’ (19.8%, 43/217), ‘not comfortable supplying MC products now but interested in doing so in the future’ (20.3%, 44/217), or ‘not comfortable supplying MC products and not wishing to do so in the future’ (6.0%, 13/217). Asymptotic Linear-by-Linear Association Tests and correspondence analyses found a significant association between ‘comfort levels’ and age, with participating pharmacists aged ≥ 45 years more comfortable supplying MC products (z = 3.25, *p* = 0.001). ‘Comfort level’ was also associated with experience, with participating pharmacists having ≥ 16 years of experience more comfortable supplying MC products (z = 2.63, *p* = 0.009). Higher ‘confidence’ in discussing customers’ enquiries about MC products was significantly associated with the male gender (z = 2.49, *p* = 0.013). Male gender (z = 3.166, *p* = 0.002) and age ≥ 45 years (z = 2.979, *p* = 0.003) were significantly associated with more support for low-dose CBD. No other significant associations were detected.

Respondents who had experience dispensing MC products (60.0%, 130/217) were presented with a list of common MC indications and asked what they believed were the three main conditions for which MC is used based on their experience. The top condition was non-cancer pain (55.3%, 120/217), followed by anxiety (29.0%, 63/217), neuropathic pain (26.3%, 57/217), chronic cancer pain (21.2%, 46/217), insomnia (15.2%, 33/217), childhood epilepsy (13.8%, 30/217), spasticity (9.2%, 20/217), ‘other’ (6.9%), depression (6.5%, 14/217) and ‘unsure’ (0.5%, 1/217).

### Pharmacists’ attitudes towards the supply of medicinal cannabis products

**Fig. 1:**
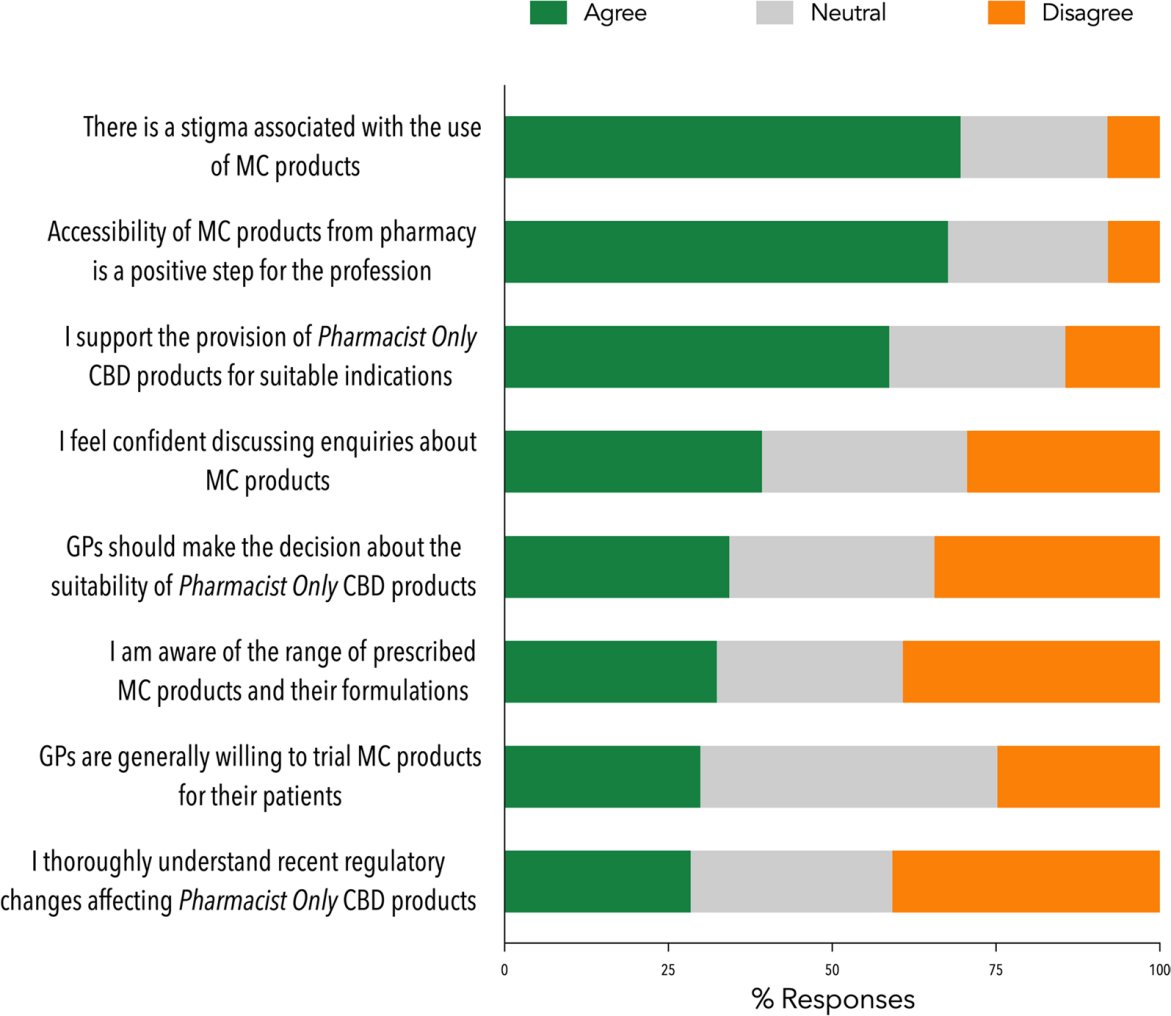
Pharmacists’ responses to statements on various aspects of the supply of medicinal cannabis and *Pharmacist Only* CBD products, n = 201, valid percentage. Abbreviation: MC: medicinal cannabis. Refer to the supplementary materials for a full copy of the survey questions

Respondents (n = 201) were asked about their attitudes towards the supply of MC and *Pharmacist Only* CBD products (Fig. 1). Overall, many respondents (67.7%, 136/201) ‘agreed’ that the accessibility of MC products from community pharmacies was a ‘positive’ step for the profession, despite 69.6% (140/201) acknowledging an ongoing stigma associated with the use of MC products. Respondents supported the provision of *Pharmacist Only* CBD products for suitable patients (58.7%, 118/201). However, only 39.3% ‘agreed’ that they felt confident discussing customers’ enquiries about MC products.

Few respondents ‘agreed’ when asked if they had a thorough understanding of the recent scheduling changes affecting *Pharmacist Only* CBD products (28.4%, 57/201), or if they were aware of the current range of MC products and formulations (32.4%, 65/201). Respondents varied in their responses when asked if they thought GPs were willing to prescribe MC products to their patients and whether GPs should decide on the suitability of *Pharmacist Only* CBD products for patients (Fig. 1).

**Fig. 2:**
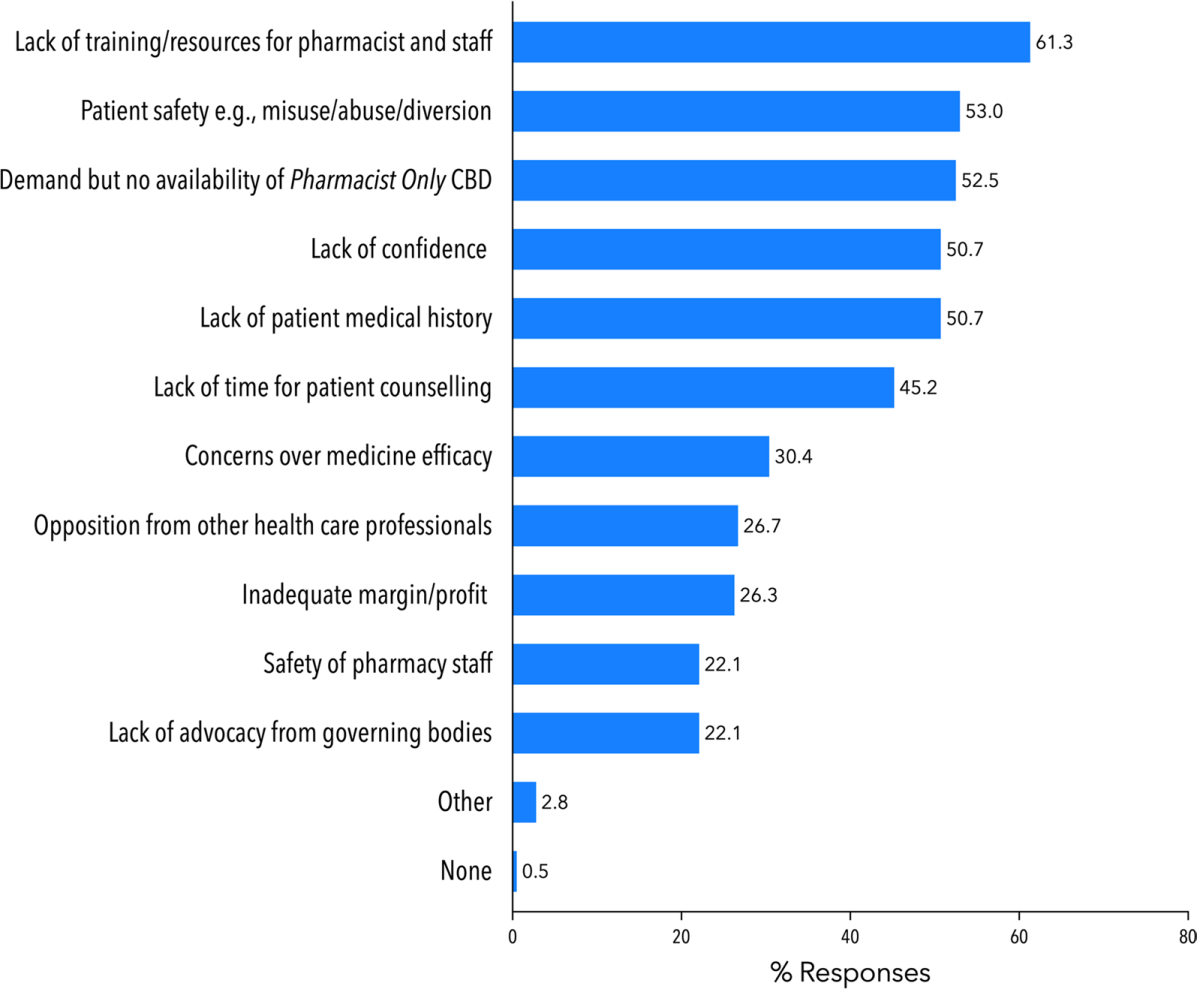
Pharmacists’ perceived barriers around the implementation of *Pharmacist Only* CBD products. Pharmacists (n = 217) were instructed to select all applicable options. Each bar represents % of total respondents (n = 217) who selected this option. Abbreviation: MC: medicinal cannabis. Refer to the supplementary material for a full copy of the survey questions

Respondents (n = 217) were presented with a list of potential barriers and asked to select all options that they believed would affect the provision of *Pharmacist Only* CBD products (Fig. 2). The most frequently selected barriers were: (1) a lack of training and resources for pharmacists and staff (61.3%, 133/217), (2) patient safety e.g., potential for misuse, abuse, diversion (53.0%, 115/217) and (3) demand, but lack of an approved *Pharmacist Only* CBD product (52.5%, 114/217).

**Fig. 3:**
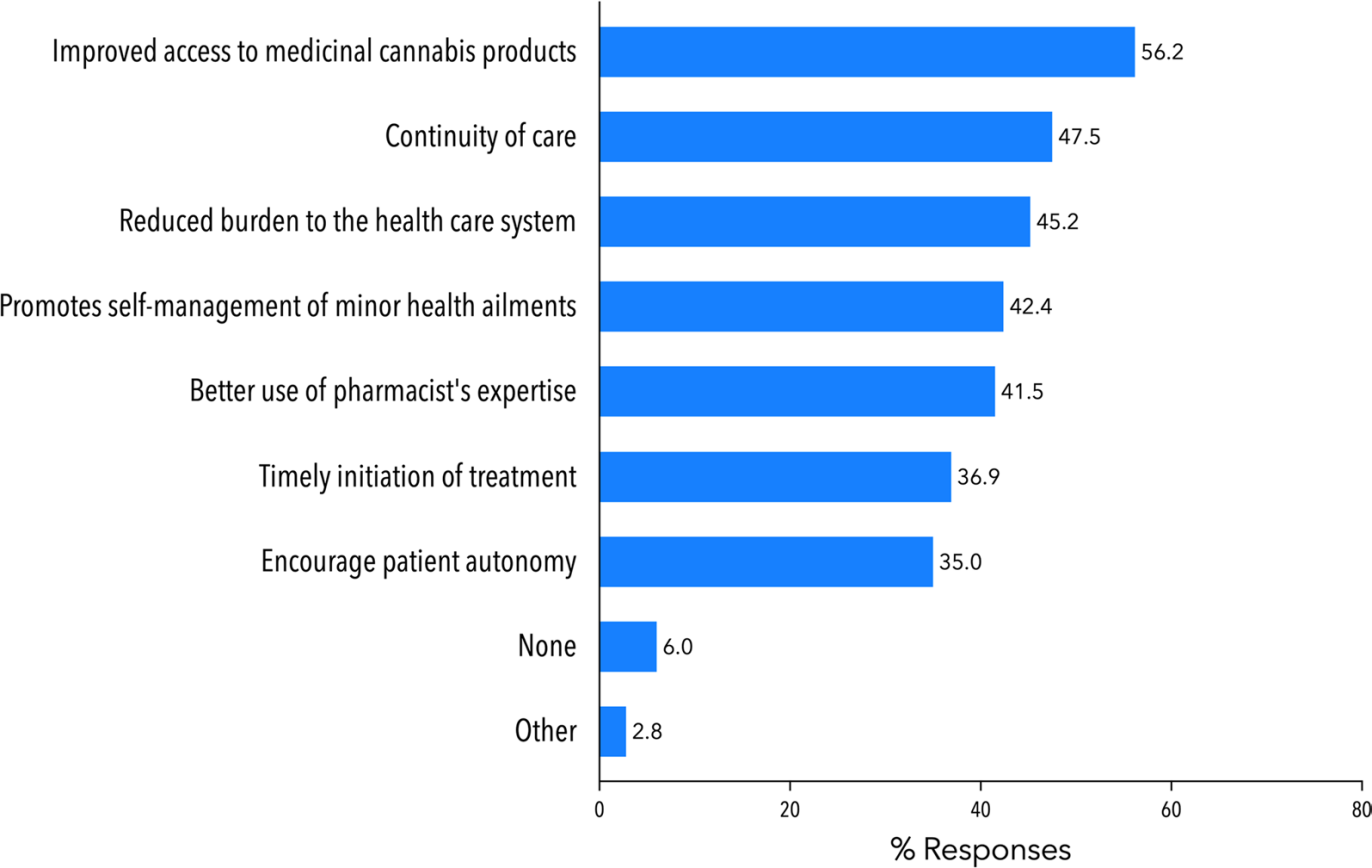
Pharmacists’ perceived benefits around the implementation of *Pharmacist Only* CBD products. Pharmacists (n = 217) were instructed to select all applicable options. Each bar represents % of total respondents (n = 217) who selected this option. Abbreviation: MC: medicinal cannabis. Refer to the supplementary material for a full copy of the survey questions

Respondents were presented with a list of perceived benefits arising from availability of *Pharmacist Only* CBD products and asked to select all options that they agreed with (Fig. 3). The most frequently selected benefits were: (1) improved access to MC products for patients (56.2%, 122/217), (2) continuity of care (47.5%, 103/217) and (3) a reduced burden to the health care system (45.2%, 98/217).

### Knowledge around medicinal cannabis products

**Fig. 4:**
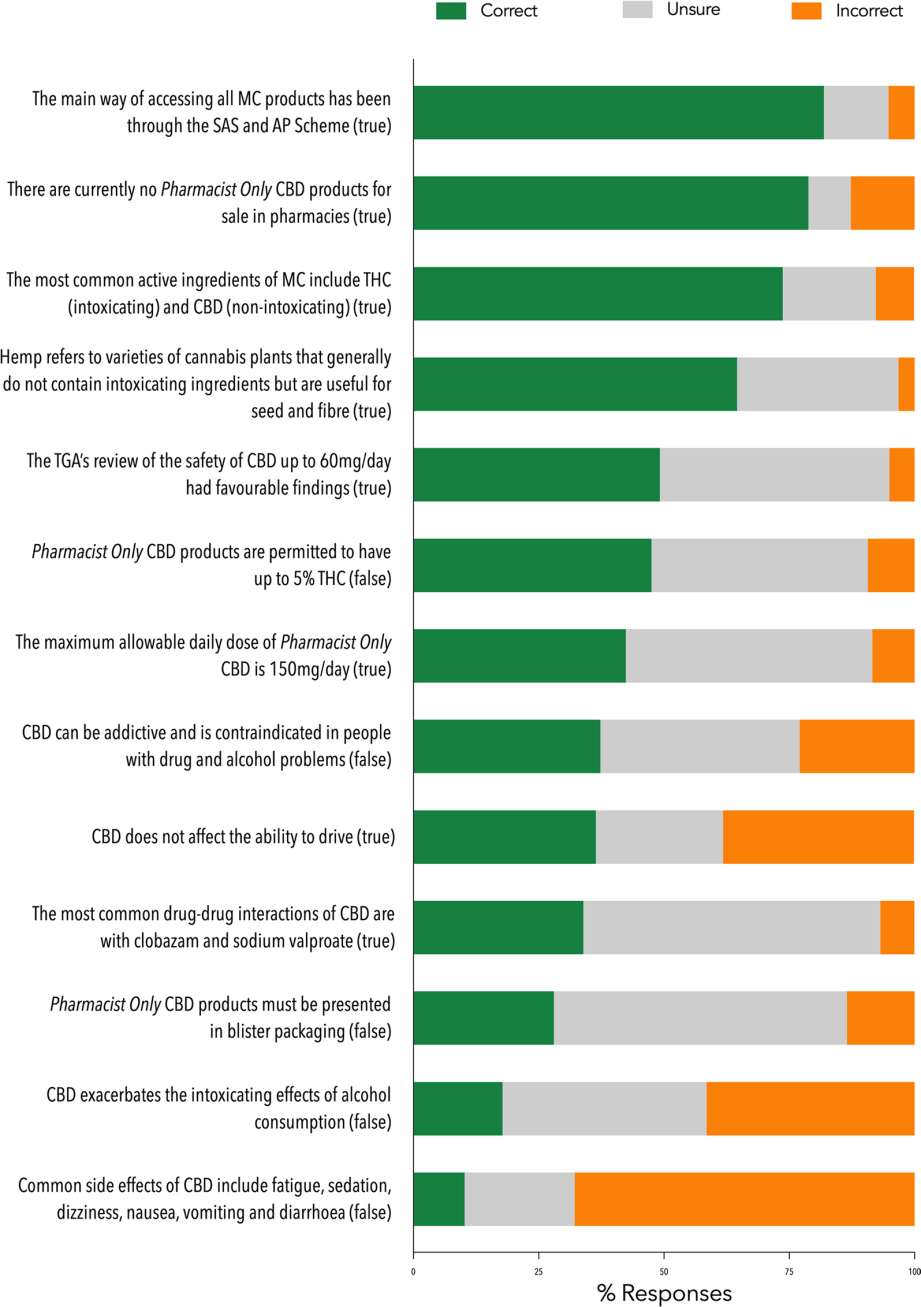
Knowledge of pharmacists around cannabis-based medicines. Figure shows percentage for each question that were answered correctly, incorrectly or as ‘unsure’ by pharmacists, n = 118, valid percentage. Correct answers provided after each question. Abbreviations: MC: medicinal cannabis; SAS: Special Access Scheme; AP Scheme: Authorised Prescriber Scheme; THC: tetrahydrocannabinol; CBD: cannabidiol; TGA: Therapeutic Goods Administration. Refer to the supplementary material for a full copy of the survey questions

Due to a technical issue, only 54.4% (118/217) respondents completed the knowledge section of the survey (Fig. 4). Only around one-third of completers (32.2%, 38/118) recorded a ‘satisfactory’ knowledge score of ≥ 60% (i.e., ≥ 8 correct out of 13 questions). The remainder had ‘low’ knowledge scores of < 60% with 21.2, 27.1 and 19.5% providing 6–7, 4–5 and 0–3 correct responses, respectively. The questions that were most commonly answered correctly involved the correct identification of: (1) the access schemes through which MC can be prescribed, (2) the current lack of registered *Pharmacist Only* CBD products and (3) the common active ingredients found in MC (81.9, 78.8 and 73.7%, correct respectively). The questions that were most commonly answered incorrectly involved CBD and adverse effects, potentiating effects with alcohol, and impacts on driving ability (67.8, 41.5 and 38.1%, incorrect respectively).

### Pharmacists’ professional education needs

Most respondents (94.2%, n = 178/189) ‘agreed’ that they required training/education on MC and *Pharmacist Only* CBD products. ‘Self-learning’ was the preferred form of training (69.3%, 131/189), followed by virtual webinars (57.7%, 109/189), pharmacy journals (47.0%, 89/189) and in-person workshops (33.3%, 63/189). Just over half of the respondents (54.5%, 103/189) ‘disagreed’ that they were provided with adequate support and information on MC and *Pharmacist Only* CBD products from their professional pharmacy organisations.

## Discussion

### Key findings

Pharmacists generally supported the accessibility of cannabis-based medicines from community pharmacies and an expanded role for pharmacists in supplying *Pharmacist Only* CBD products. Over half of the respondents had dispensed a MC product during their careers and had fielded an enquiry about MC in the past fortnight. However, only around half of the pharmacists were comfortable with supplying MC products and even fewer felt confident managing cannabis-related enquiries, despite such enquiries steadily increasing over time. Most pharmacists did not believe that they had a good understanding of recent regulatory changes affecting *Pharmacist Only* CBD products. The vast majority of participating pharmacists expressed a need for professional educational support.

### Strengths and weaknesses

This is the first survey to explore Australian pharmacists’ attitudes, beliefs and knowledge around the supply of cannabis-based medicines since legal MC access was introduced into Australia in 2016. The survey is unique in that it was conducted after the Australian Government permitted access to low-dose CBD products without a prescription, but prior to any products being registered for this purpose and therefore available in pharmacies. Since 2016, access to MC products has increased dramatically in the Australian community [42] so the survey provides key insights into the impacts of this improved availability on pharmacists. This survey is also reasonably comprehensive with 51 items scoping numerous MC aspects that are relevant to pharmacy practice. The 217 pharmacists included in this study are a small portion of Australia’s population of ~ 35,000 pharmacists [43]. However, respondents in this survey closely represented the overall demographic profile of Australia’s pharmacist workforce of whom more than half identify as female (63.0%) and one-third represent a younger age group (36.2% aged 25–34 years), or work in NSW (29.9%) [43].

This study has some limitations. The COVID-19 pandemic contributed to the difficulty in recruiting pharmacists for this survey. A technical issue with the survey led to some data not being collected for the Knowledge section of the survey. The Knowledge section of the survey was also completed unsupervised. The survey was not psychometrically tested and may be affected by recall bias. There was no way to prevent pharmacists from completing the survey more than once. The recruitment strategies may have biased the results by appealing to pharmacists who were technologically adept and had a strong interest in MC. The generalisability of the findings is limited by the relatively small sample size, meaning that the results may not entirely represent Australian pharmacists.

### Interpretation

Australian pharmacists have a key role in facilitating access to novel therapeutics and substantial recent experience with medicines being down-scheduled from *Prescription Only* to *Pharmacist Only* medicines (e.g., melatonin supply for older people with insomnia, morning-after contraceptives) [44–46]. Pharmacists value their involvement in the discourse around MC, given their essential role in supply [1]. Nonetheless, pharmacists in this survey flagged potential barriers to assisting the community with access to MC products. Few respondents felt confident discussing customers’ enquiries about MC products despite the rise in such enquiries, and only around half of the respondents felt comfortable supplying MC. Similar findings are observed in many international surveys of pharmacists around MC [1, 31–34, 47]. These results may reflect pharmacists’ need to navigate the broad range of clinical applications for cannabis-based medicines [2, 5, 42], only some of which are supported by high-quality evidence [48], and the extensive product range in Australia involving around 240 individual preparations [2, 5]. Participating pharmacists aged ≥ 45 years or with ≥ 16 years of experience were significantly more comfortable with MC supply than other pharmacists. This suggests that significant expertise in pharmacy practice aided pharmacists when managing MC supply.

Male pharmacists were found to be more supportive of *Pharmacist Only* CBD being available in pharmacies and more confident with managing MC enquiries than female pharmacists. Gender differences were also noted in a Canadian survey of pharmacists and pharmacy students around MC, whereby male pharmacists were more comfortable counselling patients around MC than their female colleagues [47]. Research suggests that women often demonstrate lower confidence than men in various contexts. While this may influence decision-making, self-reported confidence is not necessarily an accurate indicator of ability [49]. These factors must be considered in the interpretation of results.

Lack of pharmacist confidence and comfort with MC supply was underlined by ‘low’ objective knowledge; two-thirds of pharmacists who completed the Knowledge section of the survey achieved a score of < 60%. While these results may not entirely represent the whole survey cohort, many pharmacists also noted low perceived knowledge around regulatory changes affecting *Pharmacist Only* CBD products. This may reflect the fact that no *Pharmacist Only* CBD products are currently registered or marketed, meaning that pharmacists may not consider competency in this area a priority. The low levels of knowledge observed in this study are consistent with numerous surveys of pharmacists around cannabis-based medicines [31–34, 50, 51]. Indeed, the piecemeal approach to legalising MC has been identified as creating gaps in knowledge [51]. In one review, 58.9–81.9% of pharmacists were predicted to have a low perceived knowledge around MC [34], while only 5% of pharmacists in another survey of Californian pharmacists reported having ‘professional level’ knowledge around MC [33]. When *Pharmacist Only* CBD products become available, knowledge development in this area will be essential for optimal patient care and to actualise health professionals’ roles to their full potential [33, 36], particularly with pharmacists set to become the ‘frontline suppliers’ of *Pharmacist Only* CBD products.

Almost all survey respondents strongly endorsed the need for further training. This need has been repeatedly highlighted as a priority in surveys of pharmacists around MC [1, 31, 33, 34, 47, 50, 51], at times, in equally high numbers, i.e., ~ 70–90% of pharmacists surveyed [33, 34]. A significant obstacle to pharmacists’ education around cannabis-based therapy has been noted as a lack of access to reliable resources and training activities [34, 50, 51]. Currently, an array of MC resources, guidelines, and policies are available from Australian government organisations such as the TGA and pharmacy-specific organisations [4, 48, 52–56]. However, these resources are lengthy and have not been streamlined or tailored to pharmacy practice. Furthermore, resources for pharmacists, specifically around managing *Pharmacist Only* CBD products, are scarce. Indeed, most pharmacists did not believe they were provided adequate support around cannabis-based medicines and *Pharmacist Only* CBD products from professional organisations, indicating that a call to action is required in this space. Educational initiatives have accompanied the down-scheduling of other *Pharmacist Only* products in Australia and resulted in a safe supply of down-scheduled products [44–46, 57].

This survey highlights a number of other concerns that would be beneficial to mitigate. Firstly, the stigma associated with the use of MC was flagged by most pharmacists in this survey, in line with previous surveys [34, 51]. The stigma around MC may arise from the confusion between medicinal and recreational cannabis [34]. Stigma can lead to marginalisation, disempowerment and poor health outcomes for patients utilising these products [51, 58, 59]. Secondly, pharmacists demonstrated concerns about the potential for misuse, abuse, diversion and the effects on driving with *Pharmacist Only* CBD products. These issues have been flagged in surveys of MC and may highlight concerns around THC that some pharmacists also mistakenly attribute to CBD [1, 31, 34]. Indeed, the 2018 World Health Organization Expert Committee on Drug Dependence deemed CBD to have a good safety profile with a low potential for adverse effects [60], reaffirmed by a recent review of low dose CBD safety and efficacy [61]. Thirdly, 6% of pharmacists in this survey noted having no interest in supplying MC products. However, current resources do not provide specific guidance or support for pharmacists who may wish to opt-out of supply [4, 48, 52–56]. These concerns are important to address as patients may be left to navigate the convoluted area of cannabis-based therapy alone, increasing the risk of unsafe practices [51]. Government and professional bodies will play a critical role in the education and support of pharmacists. A clear and strategic pathway to clinical competence is essential before *Pharmacist Only* CBD products become widely available, whether pharmacists decide to be involved with supply or not.

### Further research

Further research may involve developing of training and educational resources around MC and *Pharmacist Only* CBD products. The ‘pain-points’ highlighted in this survey may provide insights into the content of such initiatives, which should be evidence-based, relevant and specific to pharmacy practice [62]. The significant workload-related constraints on pharmacists’ time should be carefully considered [62], as should the flexibility and variety of training activities [62, 63]. Indeed, this survey and prior surveys demonstrated that pharmacists had clear preferences for learning activities [31, 34, 51]. These resources should guide all pharmacists, including those who opt out of MC supply.

## Conclusion

This survey explored Australian pharmacists’ experience, knowledge and attitudes towards MC and their preparedness to supply low-dose CBD products. This survey was conducted at a time of accelerating demand for MC products in Australia, just before *Pharmacist Only* CBD products become available in pharmacies. Pharmacists generally support their role in the supply of *Pharmacist Only* CBD products and have critical fundamental skills and experience to aid in supply. However, pharmacists require further training to optimise their role to its full potential. This survey provides a unique perspective on the management of MC in Australia and complements prior research in this area. These findings may be relevant to other jurisdictions where improved community access to medicinal cannabis products is being contemplated.

## Electronic supplementary material

Below is the link to the electronic supplementary material.

Supplementary Material 1
