# Supplementary material for: A cross-sectional survey exploring the knowledge, experiences and attitudes of Australian pharmacists toward medicinal cannabis
Source: Int J Clin Pharm. 2022 Nov 29;45(2):375–86. doi: 10.1007/s11096-022-01519-z (PMC9708126; doi:10.1007/s11096-022-01519-z)
Supplement: Supplementary file 1 — Supplementary Material 1 [file 11096_2022_1519_MOESM1_ESM.pdf]

A Cross-Sectional Survey Exploring the Knowledge, Experiences and Attitudes of Australian Pharmacists Toward Medicinal Cannabis

Zeeta Bawa [1-3], Bandana Saini [4,5], Danielle McCartney [1-3], Miguel Bedoya Perez[1-3], Andrew J. McLachlan [4], Iain S. McGregor [1-3]

[1] The University of Sydney, Lambert Initiative for Cannabinoid Therapeutics, Sydney, New South Wales, Australia
[2] The University of Sydney, Brain and Mind Centre, Sydney, New South Wales, Australia
[3] The University of Sydney, Faculty of Science, School of Psychology, Sydney, New South Wales, Australia
[4] The University of Sydney, Sydney Pharmacy School, Sydney, New South Wales, Australia
[5] Woolcock Institute of Medical Research, Sydney, New South Wales, Australia

***Correspondence:** Professor Iain S. McGregor; contact: iain.mcgregor@sydney.edu.au

Online Resource 1

Community Pharmacists and Cannabidiol (CBD) Online Survey
Project Number: 2021/149

PARTICIPANT QUESTIONNAIRE

Section 1: The following section collects anonymous demographic information. We ask this to explore if professional education needs vary with experience or locality.

1. What is your age?

☐ 18-24 years
☐ 25-34 years
☐ 35-44 years
☐ 45-54 years
☐ 55-64 years
☐ 65+ years

2. How would you describe your gender?

☐ Male
☐ Female
☐ Other
☐ Prefer not to answer

3. Where in Australia do you practice?

☐ New South Wales
☐ Victoria
☐ South Australia
☐ Western Australia
☐ Queensland
☐ Australian Capital Territory
☐ Northern Territory
☐ Tasmania

4. **What is your <u>main role</u> in community pharmacy? (Select all that apply*)***

- ☐ Intern pharmacist
- ☐ Pharmacist employee
- ☐ Pharmacist-in-Charge
- ☐ Pharmacy owner
- ☐ Locum pharmacist
- ☐ AACP Accredited Pharmacist
- ☐ Other, please specify

5. **How many years of experience do you have as a pharmacist?**

- ☐ Less than 1 year
- ☐ 1-5 years
- ☐ 6-10 years
- ☐ 11-15 years
- ☐ 16-19 years
- ☐ 20 or more years

6. **What is the setting of your pharmacy? (Select the best option)**

- ☐ Shopping centre
- ☐ Medical centre
- ☐ Shopping strip
- ☐ Stand-alone pharmacy
- ☐ Other, please specify_________

7. **How would you best describe the type of pharmacy where you mainly work? (Select the best option)**

- ☐ Chain or franchise
- ☐ Independent
- ☐ Other, please specify__________

8. **What best describes the location of your pharmacy? (Select the best option)**

- ☐ Major metro city centre
- ☐ Major suburban city centre
- ☐ Regional town
- ☐ Rural or remote town
- ☐ Other, please specify___________

9. **What is the post code of the main pharmacy where you work?**

- ☐ Prefer not to answer
- ☐ Comfortable to answer: ____________

10. **On an average weekday in your pharmacy, what is the total number of prescriptions dispensed? (Select the best option)**

- ☐ 0-50
- ☐ 51-100
- ☐ 101-150
- ☐ Over 150
- ☐ Unsure

Section 2: This section explores your pharmacy's current involvement in supply of prescribed medicinal cannabis products, if any. Please note that this includes products containing cannabidiol (CBD)-only, tetrahydrocannabinol (THC)-only and products containing combinations of THC and CBD.

11. Regardless of whether you have dispensed medicinal cannabis products or not, what are your feelings towards supply? (Select the best option)

- ☐ I am not comfortable supplying medicinal cannabis products and do not wish to in the future
- ☐ I am not comfortable supplying medicinal cannabis products now but am interested in supply in the future
- ☐ I am neither comfortable nor uncomfortable supplying medicinal cannabis products
- ☐ I am comfortable supplying medicinal cannabis products and wish to in the future

12. What best describes your experience in relation to the supply of medicinal cannabis products? (Select the best option)

- ☐ My pharmacy does not supply medicinal cannabis products and I have never dispensed them
- ☐ My pharmacy does supply medicinal cannabis products but I have never dispensed them
- ☐ I have dispensed medicinal cannabis products, but not in the past month
- ☐ I have dispensed medicinal cannabis products in the past month

Questions 13 – 20 will only appear if medicinal cannabis products were dispensed as per Question 11

13. In the past two months, approximately how many medicinal cannabis prescriptions did <u>you</u> personally dispense?

- ☐ 1-4
- ☐ 5-9
- ☐ 10-19
- ☐ 20 or more
- ☐ Unsure

14. In the past month, approximately how many medicinal cannabis prescriptions did <u>your pharmacy</u> dispense?

- ☐ 1-4
- ☐ 5-9
- ☐ 10-19
- ☐ 20 or more
- ☐ Unsure

15. Are the majority of medicinal cannabis prescriptions repeats or new prescriptions?

- ☐ Repeats
- ☐ New prescriptions
- ☐ Unsure

16. Epidiolex™ is currently the only prescription <u>CBD-only</u> product registered on the Australian Register of Therapeutic Goods (ARTG). Has your pharmacy ever dispensed a prescription for this medication?

- ☐ Yes
- ☐ No
- ☐ Unsure

17. Sativex ™ is currently the only prescription <u>THC/CBD containing product</u> registered on the Australian Register of Therapeutic Goods (ARTG). Has your pharmacy ever dispensed a prescription for this medication?

- ☐ Yes
- ☐ No
- ☐ Unsure

18. If you have insights into why your customers are using medicinal cannabis products, please select up to three indications below. (Select top three)

- ☐ I do not have insights
- ☐ Anxiety
- ☐ Insomnia
- ☐ Chronic non-cancer pain (e.g. arthritis, back pain, neck pain)
- ☐ Chronic cancer pain
- ☐ Neuropathic pain
- ☐ Depression
- ☐ Childhood epilepsy
- ☐ Spasticity from conditions such as multiple sclerosis
- ☐ Other (please specify): ________________

19. Are you aware of any customers who have stopped taking prescription medicinal cannabis products because of adverse effects?

- ☐ Yes
- ☐ No
- ☐ Unsure

20. In the past 2 weeks at your pharmacy, approximately how many customers have asked you about medicinal cannabis, including CBD products? (Select the best option)

- ☐ Nil
- ☐ 1-4
- ☐ 5-9
- ☐ 10-19
- ☐ 20 or more

21. In the past 3 months at your pharmacy, there has been a steady increase in customers asking about medicinal cannabis products, including CBD products. (Select the best option)

- ☐ True
- ☐ False
- ☐ Unsure

Section 3: The following section explores professional education needs specifically relating to CBD products. CBD is the non-intoxicating component of cannabis. As you would be aware, low dose

CBD products are legally available as *Pharmacist Only* Schedule 3 (S3) products in community pharmacies as of 1 February 2021.

22. **The maximum allowable daily dose of Schedule 3 CBD is 150mg/day (Select the best option)**

- ☐ True
- ☐ False
- ☐ Unsure

23. **There are currently no *Pharmacist Only* S3 registered CBD products on the Australian Register of Therapeutic Goods (ARTG) for sale in community pharmacies. (Select the best option)**

- ☐ True
- ☐ False
- ☐ Unsure

24. ***Pharmacist Only* S3 CBD products are permitted to have up to 5% THC content. (Select the best option)**

- ☐ True
- ☐ False
- ☐ Unsure

25. ***Pharmacist Only* S3 CBD products must be presented in blister packaging. (Select the best option)**

- ☐ True
- ☐ False
- ☐ Unsure

26. **Up until now, the main way of accessing medicinal cannabis products including CBD products has been under the TGA Special-Access Scheme B or Authorised Prescriber Scheme**

- ☐ True
- ☐ False
- ☐ Unsure

27. **The two most common active ingredients of medicinal cannabis include Δ-9 tetrahydrocannabinol (THC) which is intoxicating; and cannabidiol (CBD) which is non-intoxicating. (Select the best option)**

- ☐ True
- ☐ False
- ☐ Unsure

28. **The TGA's review of the safety of low dose CBD (up to 60mg/day) had favourable findings. (Select the best option)**

- ☐ True
- ☐ False
- ☐ Unsure

29. **Common side effects of CBD include fatigue, sedation, dizziness and gastrointestinal upsets including nausea, vomiting and diarrhoea. (Select the best option)**
- ☐ True
- ☐ False
- ☐ Unsure

30. **The most common drug-drug interactions of CBD are with clobazam and sodium valproate (Select the best option)**
- ☐ True
- ☐ False
- ☐ Unsure

31. **Hemp refers to varieties of cannabis plants that generally do not contain intoxicating ingredients but are useful for seed and fibre. (Select the best option)**
- ☐ True
- ☐ False
- ☐ Unsure

32. **CBD does not affect the ability to drive. (Select the best option)**
- ☐ True
- ☐ False
- ☐ Unsure

33. **CBD exacerbates the intoxicating effects of alcohol consumption. (Select the best option)**
- ☐ True
- ☐ False
- ☐ Unsure

34. **CBD can be addictive and is contraindicated in people with drug and alcohol problems. (Select the best option)**
- ☐ True
- ☐ False
- ☐ Unsure

Section 4: The following section explores beliefs, feelings and levels of confidence with respect to medicinal cannabis products and low dose CBD in community pharmacy

35. **As a pharmacist, do you believe that the accessibility of medicinal cannabis products from community pharmacy is a positive or negative step for the profession?**
- ☐ Very positive
- ☐ Positive
- ☐ Neutral
- ☐ Negative
- ☐ Very Negative

36. Please describe why the accessibility of medicinal cannabis products from community pharmacy is a positive or negative step for the profession:

____

37. GPs have generally been willing to trial medicinal cannabis products on their patients if suitable. (Select the best option)

- ☐ Strongly Agree
- ☐ Agree
- ☐ Neutral
- ☐ Disagree
- ☐ Strongly Disagree

38. I feel confident discussing customer's enquiries about medicinal cannabis products. (Select the best option)

- ☐ Strongly Agree
- ☐ Agree
- ☐ Neutral
- ☐ Disagree
- ☐ Strongly Disagree

39. There is a stigma associated with the use of medicinal cannabis products. (Select the best option)

- ☐ Strongly Agree
- ☐ Agree
- ☐ Neutral
- ☐ Disagree
- ☐ Strongly Disagree

40. I am aware of the current range of prescribed medicinal cannabis products and their formulations. (Select the best option)

- ☐ Strongly Agree
- ☐ Agree
- ☐ Neutral
- ☐ Disagree
- ☐ Strongly Disagree

41. I support the provision of *Pharmacist Only* S3 CBD products to my customers for suitable indications. (Select the best option)

- ☐ Strongly Agree
- ☐ Agree
- ☐ Neutral
- ☐ Disagree
- ☐ Strongly Disagree

42. I would rather a GP make the decision about the suitability of *Pharmacist Only* S3 CBD products for my customers. (Select the best option)

- ☐ Strongly Agree
- ☐ Agree
- ☐ Neutral

- ☐ Disagree
- ☐ Strongly Disagree

43. I have a thorough understanding of the TGA's recent scheduling changes affecting low dose CBD products. (Select the best option)

- ☐ Strongly Agree
- ☐ Agree
- ☐ Neutral
- ☐ Disagree
- ☐ Strongly Disagree

44. As a pharmacist, what do you believe are the challenges that your profession will face in implementing *Pharmacist Only* S3 CBD products? (Select all that apply)

- ☐ Inadequate margin/profit in selling products
- ☐ Opposition from other health care professionals
- ☐ Lack of patient medical history
- ☐ Patient safety e.g., misuse/abuse/diversion
- ☐ Safety of pharmacy staff
- ☐ Lack of time for patient counselling
- ☐ Lack of advocacy from governing bodies
- ☐ Lack of training/resources for pharmacist and staff
- ☐ Lack of confidence
- ☐ Concerns over medicine efficacy
- ☐ Demand for low dose CBD but no registered S3 products currently available
- ☐ None of the above
- ☐ Others, please specify_______________

45. The benefits of *Pharmacist Only* S3 access to CBD include (select all that apply)

- ☐ Continuity of care
- ☐ Timely initiation of treatment
- ☐ Promotes self-management of minor health ailments
- ☐ Encourage patient autonomy
- ☐ Reduced burden to the health care system
- ☐ Better use of pharmacist's expertise
- ☐ Improved access to medicinal cannabis products
- ☐ None of the above
- ☐ Others, please specify:___________________-

Section 5: This section explores what your needs are, as a community pharmacist, in relation to the scheduling changes of CBD products

46. I am familiar with where to access resources to support my professional development on medicinal cannabis and the recent *Pharmacist Only* S3 CBD scheduling changes (Select the best option)

- ☐ Strongly Agree

- ☐ Agree
- ☐ Neutral
- ☐ Disagree
- ☐ Strongly Disagree

47. I believe that pharmacy curriculums at university should include the topic of cannabis as a therapeutic area (Select the best option)

- ☐ Strongly Agree
- ☐ Agree
- ☐ Neutral
- ☐ Disagree
- ☐ Strongly Disagree

48. I require training/education on medicinal cannabis products and *Pharmacist Only* S3 CBD products (Select the best option)

- ☐ Strongly Agree
- ☐ Agree
- ☐ Neutral
- ☐ Disagree
- ☐ Strongly Disagree

49. I have been provided with adequate support and information on medicinal cannabis products and *Pharmacist Only* S3 CBD from my pharmacy and professional pharmacy bodies. (Select the best option)

- ☐ Strongly Agree
- ☐ Agree
- ☐ Neutral
- ☐ Disagree
- ☐ Strongly Disagree

50. In the last TWO years, do your recall having undertaken any continuing professional development (CPD) on the topic of medicinal cannabis products? (Select the best option)

- ☐ Yes
- ☐ No
- ☐ I can’t remember

51. If you were to choose training/education activities, please indicate which would benefit you most? (Select all that apply)

- ☐ Self-learning material and resources
- ☐ Virtual webinars or workshops
- ☐ Face to face presentations or workshops
- ☐ Educational pieces in various pharmacy journals

If you have any further comments, thoughts or suggestions regarding CBD and community pharmacists, please leave below:

